# Supplementary material for: What Mothers Know about Newborn Bloodspot Screening and the Sources They Use to Acquire This Knowledge: A Pilot Study in Flanders
Source: Children (Basel). 2023 Sep 18;10(9):1567. doi: 10.3390/children10091567 (PMC10529094; doi:10.3390/children10091567)
Supplement: Supplementary file 1 [file children-10-01567-s001.zip › children-2602886-supplementary.pdf]

Table S1. The final questionnaire (in Dutch)

Opinies/Kennisvragen, gesloten (ja/nee)
De ziektes die de hielprik opspoort, hebben zeer ernstige gevolgen als ze niet behandeld worden.
De kans dat een kind één van de 'hielprik'ziektes heeft is zeer klein.
Bij de hielprik wordt wat bloed afgenomen uit de hiel van het kind
Als de uitslag van de hielprik afwijkend is, dan is er pas zekerheid over een ziekte als er meer onderzoek is gedaan in het ziekenhuis.
Een goede uitslag van de hielprik is een garantie dat het kind helemaal gezond is.
De hielprik is een betrouwbare test, omdat kinderen met één van die ziektes bijna altijd worden opgespoord.
Als de uitslag van de hielprik onduidelijk is, dan is een tweede hielprik nodig.
De hielprik is verplicht voor elke pasgeboren baby.
Iemand die gezond is, kan drager zijn van een erfelijke ziekte.
Als de uitslag van de hielprik GOED is, dan ontvangen de ouders GEEN bericht
Na het onderzoek worden de bloeddruppels direct vernietigd.
Bent U op de hoogte van de recente uitbreiding van de Guthrie test ?
**Kennisvragen, multiple choice, inclusief open vragen**
Van wie heeft U informatie gekregen over de hielprikscreening (U kan meerdere antwoorden aanduiden) (*vroedvrouw, verpleegkundige, gynaecoloog, huisarts, infomoment over zwangerschap, vrienden of kennissen, website 'aangeboren.bevolkingsonderzoek.be, televisie of radio, kranten of tijdschriften, infobrochure, social media, ik heb geen informatie gekregen, andere*)
Welke bron van informatie over de hielprik was voor U het belangrijkst (U kan meerdere antwoorden aanduiden) (*vroedvrouw, verpleegkundige, gynaecoloog, huisarts, infomoment over zwangerschap, vrienden of kennissen, website 'aangeboren.bevolkingsonderzoek.be, televisie of radio, kranten of tijdschriften, infobrochure, social media, ik heb geen informatie gekregen, andere*)
Als U een folder over de hielprik heeft gekregen, heeft U die bekeken (*ja helemaal gelezen, ja deels gelezen, ja in gebladerd maar niet in detail doorgelezen, nee ik kende de folder al, nee ik wist al genoeg over de hielprik, niet van toepassing want folder niet gekregen*) ?
Heeft U zelf actief andere informatie gezocht over de hielprik (*ja via de website 'aangeboren.bevolkingsonderzoek.be, ja via internet maar andere websites, andere folders boeken of tijdschriften gelezen, ja ik ben met anderen gaan praten, ja anders namelijk*) ?
Wat vindt U in het algemeen van de informatie dit U heeft gekregen vanuit de gezondheidszorg (0-5, 0 is de slechte score) (duidelijk, tijdig, voldoende, nuttig)
